# Vimentin in Bacterial Infections

**DOI:** 10.3390/cells5020018

**Published:** 2016-04-18

**Authors:** Tim N. Mak, Holger Brüggemann

**Affiliations:** Department of Biomedicine, Aarhus University, 8000 Aarhus C, Denmark; timnam@gmail.com

**Keywords:** vimentin, intermediate filament, innate immune signaling, pattern recognition receptor, NOD2, NF-kB, reactive oxygen species, intracellular pathogen, bacterial infection

## Abstract

Despite well-studied bacterial strategies to target actin to subvert the host cell cytoskeleton, thus promoting bacterial survival, replication, and dissemination, relatively little is known about the bacterial interaction with other components of the host cell cytoskeleton, including intermediate filaments (IFs). IFs have not only roles in maintaining the structural integrity of the cell, but they are also involved in many cellular processes including cell adhesion, immune signaling, and autophagy, processes that are important in the context of bacterial infections. Here, we summarize the knowledge about the role of IFs in bacterial infections, focusing on the type III IF protein vimentin. Recent studies have revealed the involvement of vimentin in host cell defenses, acting as ligand for several pattern recognition receptors of the innate immune system. Two main aspects of bacteria-vimentin interactions are presented in this review: the role of vimentin in pathogen-binding on the cell surface and subsequent bacterial invasion and the interaction of cytosolic vimentin and intracellular pathogens with regards to innate immune signaling. Mechanistic insight is presented involving distinct bacterial virulence factors that target vimentin to subvert its function in order to change the host cell fate in the course of a bacterial infection.

## 1. Introduction

The type III intermediate filament protein vimentin (VIM) is a major constitutive protein of intermediate-sized filaments present in the cytoskeleton. As such it is involved in maintaining cell shape and integrity, thereby also supporting the anchorage of cell organelles such as the nucleus, endoplasmic reticulum, and mitochondria. Cells of mesenchymal origin such as endothelial cells express vimentin, but expression of vimentin in various cell types and different developmental stages is dynamic and subject to regulation involving post-translational modifications and intracellular proteases. Vimentin is also expressed in cells undergoing physiological or pathological changes, for instance in the process of epithelial-mesenchymal transition (EMT), during embryogenesis, wound healing, or metastasis [1,2,3,4].

Besides its function as a cytosolic protein, several studies reported a role of cell surface-located and extracellular vimentin [5]. Here, we highlight the main findings from investigations of the significance of vimentin in bacterial infections.

## 2. Interactions of Vimentin with Gram-Negative Bacterial Species

Several Gram-negative pathogens such as the Enterobacteriaceae *E. coli* and *Salmonella enterica* have been found to interact with vimentin inside and on the eukaryotic cell surface (Table 1).

**Figure 1:**
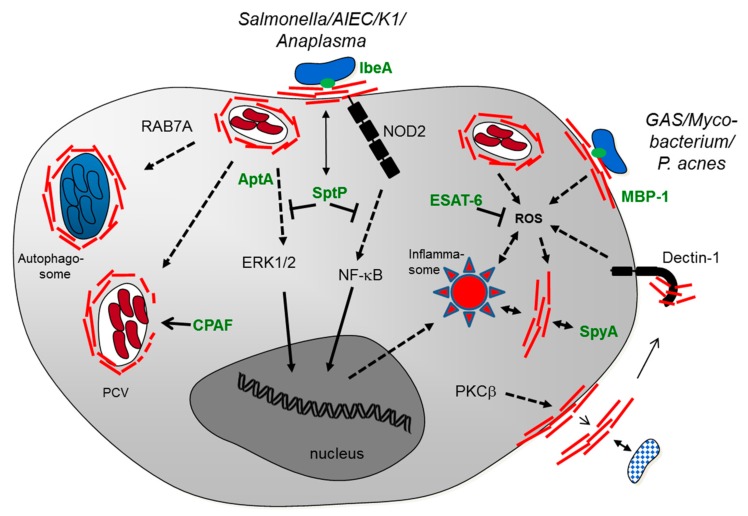
Tentative scheme of the cellular interactions of vimentin with bacterial pathogens. Cell surface-located vimentin (red lines) is involved in binding of the pathogen and subsequent invasion. A variety of intracellular vimentin-pathogen interactions is depicted, mainly occurring in human or murine macrophages. Cytosolic vimentin (red lines) is closely associated to the pathogen-containing vacuole (PCV) and to the autophagosome. The interaction of vimentin with innate immune signaling involves the pattern recognition receptors dectin-1, NOD2, and NLPR3 (inflammasome). PPR-mediated innate signaling further results in activation of MAP kinases (ERK1/2) and NF-κB. Pathogen-triggered intracellular reactive oxygen species (ROS) production is associated with vimentin upregulation. A few bacterial factors have so far been identified that can mediate vimentin binding (IbeA, MBP-1) or interfere with vimentin functionality (SptP, SpyA, CPAF, AptA; see Table 1 for their origin), possibly by proteolysis or post-translational modification of vimentin. Phosphorylation of vimentin is associated with vimentin secretion that might be involved in pathogen trapping and killing (indicated in bottom right hand corner). Further abbreviations: AIEC, adherent-invasive *E. coli*; K1, *E. coli* K1; GAS, Group A streptococci; *P. acnes*, *Propionibacterium acnes*; PKCβ, Protein kinase C beta; RAB7A, Ras-related protein Rab-7a.

First, it was shown for *E. coli* K1 strains responsible for human meningitis in neonates that they can bind to vimentin on the surface of brain microvascular endothelial cells (BMEC) [6]. This interaction is mediated by the *E. coli* virulence factor IbeA. The IbeA-vimentin interaction is required for invasion of meningitic *E. coli* into BMEC cells and involves vimentin phosphorylation and subsequent signaling via ERK1/2 (extracellular-signal-regulated kinases) and NF-κB (nuclear factor kappa-light-chain-enhancer of activated B cells), a master regulator of the immune response to infection [6,7,8] (see also Figure 1).

Other *E. coli* strains can interact with vimentin on the host cell surface, in particular adherent-invasive *E. coli* strains (AIEC) involved in inflammatory bowel diseases [9,10]. Vimentin acts as a surface-attached receptor of AIEC. In this context, it was also shown that vimentin acts as a Nucleotide-binding oligomerization domain-containing protein 2 (NOD2)-interacting protein, an intracellular pattern recognition receptor (PRR), recognizing bacterial peptidoglycan fragments [11]. This has led to the hypothesis that vimentin mediates interaction of NOD2 with the bacterial pathogen, resulting in its activation and a subsequent inflammatory response via NF-κB signaling. Thus, accumulating evidence suggests an important role of vimentin in recognizing gastrointestinal *E. coli* and mediating innate immune signaling. In line with this, decreased intestinal disease, *i.e.*, significantly less gut inflammation, is observed in VIM knockout mice compared to wildtype mice when challenged with *E. coli* [9].

For another intestinal pathogen, *Salmonella enterica* (serovar Typhimurium), it has been shown that the infection can remodel the vimentin network [12,13]. Vimentin is recruited to the membrane ruffles stimulated by *Salmonella*. The initial bacterial impact on the host cell results in cytoskeleton rearrangements and pro-inflammatory signaling mediated by MAP (mitogen-activated protein) kinases (*i.e.*, ERK1/2). Interestingly, SptP, an effector protein of *Salmonella* that is secreted via the type III secretion system, is able to reverse the changes to the host cell membrane and inhibits pro-inflammatory signaling [12]. The study of Murli *et al.* indicates that vimentin is a potential substrate for the tyrosine phosphatase domain of SptP. The authors proposed that tyrosine-phosphorylated vimentin serves as a scaffold for signaling complexes required for *Salmonella*-induced MAP kinase activation. SptP-dependent dephosphorylation of vimentin could thus inhibit signaling transduction events mediated or supported by phosphorylated vimentin. In agreement with this model, (tyrosine-)phosphorylated vimentin has been observed in MAP kinase signaling complexes after stimulation in different cell types [26,27]. Another study has shown that *Salmonella* induces the formation of aggresome-like structures, characterized by remodeled vimentin and cytokeratin networks in epithelial cells and macrophages [13]. Moreover, the presence of vimentin cages around *Salmonella*-containing vacuoles in the host cell cytosol maintains the vacuoles in close vicinity to the nucleus. The significance of this finding remains to be determined.

*Anaplasma phagocytophilum* is an obligate intracellular bacterium that can cause human granulocytic anaplasmosis, which is a tick-borne rickettsial disease. The bacterium specifically invades neutrophils. The intracellular *A. phagocytophilum*-containing vacuole is able to reorganize and recruit vimentin [14]. In particular, *A. phagocytophilum* utilizes the virulence factor AptA (*A. phagocytophilum* toxin A) to interact with vimentin, resulting in ERK1/2 activation (see also Figure 1). This suggests that the bacterium can subvert the vimentin network to modulate host immune signaling during infection.

The obligate intracellular pathogen *Chlamydia trachomatis* resides in membrane-bound vacuoles (“inclusions”). The bacterium remodels the cytoskeleton network including vimentin filaments, to form a dynamic scaffold that provides structural stability to the inclusion [15]. Interestingly, a secreted chlamydial protease CPAF (*Chlamydia* protease-like activity factor), an important factor for the full replicative potential of *C. trachomatis*, can modify the vimentin scaffold [15,16]. It has been proposed that the CPAF activity increases the inclusion’s flexibility and facilitates bacterial replication. It was further shown that CPAF cleaves vimentin, which is needed to sustain a functional, protective cage surrounding the intracellular vacuole [16]. CPAF inhibition leads to the loss of inclusion integrity, and eventually cell death. More recently, the role of CPAF has been re-examined by using CPAF loss-of-function mutants [17]. Vimentin has been confirmed as a *bona fide* target of CPAF. However, this study suggested that CPAF plays a role late in infection and that CPAF-mediated vimentin proteolysis correlates with a loss of inclusion membrane integrity. Whether vimentin stabilizes or destabilizes the *Chlamydia* inclusion might depend on different filament sizes of vimentin as well as (different) post-translational modifications.

## 3. Interactions of Vimentin with Gram-Positive Bacterial Species

Several interactions of Gram-positive bacterial species with vimentin have been investigated (Table 1 and Figure 1). The pathogen *Streptococcus pyogenes*, a prominent member of Group A streptococci (GAS), is responsible for invasive infections, including necrotizing fasciitis and septicemia. The pathogen binds to vimentin, in particular on injured skeletal-muscle cells where vimentin is upregulated following injury [18]. Another study has further shown that the pathogen can target the vimentin network, mediated by the bacterial toxin SpyA, which is a C3 family ADP-ribosyltransferase [19]. SpyA ADP-ribosylates target proteins, including actin and vimentin. The latter seems to be the preferred substrate of SpyA. ADP-ribosylation by SpyA inhibits vimentin filament formation [19]. Moreover, Icenogle *et al.* have shown that expression of SpyA in HeLa cells results in the collapse of the vimentin cytoskeleton. The exact cellular consequences of SpyA in the course of a GAS infection are unclear. It can be speculated that modification and disruption of the vimentin cytoskeleton may be important for bacterial dissemination or represents a molecular mechanism by which GAS inhibit the healing of lesions. With regard to the fate of GAS-infected cells, another study has shown a different outcome: SpyA is able to trigger cell death in macrophages by activating the inflammasome, a macromolecular complex that regulates early inflammatory responses of the innate immune system [20]. This results in restriction of bacterial growth and attenuation of disease progression. Interestingly, another recent study has shown that inflammasome activation, resulting in maturation of the inflammatory cytokine IL-1β, is mediated by vimentin [28]. Decreased active IL-1β levels are observed in vimentin-knockdown macrophages. Moreover, vimentin directly interacts with a component of the inflammasome, the pattern recognition receptor NLPR3 (nucleotide-binding domain, leucine-rich-containing family, pyrin domain-containing-3 OR NOD-like receptor protein 3). Taking the two studies [20,28] together, we speculate that SpyA-mediated manipulation of vimentin might be involved in activating the NLPR3 inflammasome. Future work has to be carried out to investigate the exact role of vimentin during GAS infection and the significance of host cell tropism, *i.e.*, differential cell responses in injured muscle cells compared with macrophages.

Several interactions of host vimentin have been described with mycobacterial species. First, it has been observed that vimentin expression is significantly upregulated on the surface of *Mycobacterium tuberculosis*-infected monocytes, compared with uninfected cells [21]. It has been further shown that vimentin binds to the NKp46 receptor of natural killer (NK) cells, suggesting that *M. tuberculosis*-infected monocytes are targeted by NK cells via vimentin-NKp46 interaction. A recent study has reported a different outcome of *M. tuberculosis* infection: here vimentin is downregulated in *M. tuberculosis*-infected macrophages compared to infections with an avirulent *M. tuberculosis* strain [22]. The apparent discrepancy of the two studies can be explained by the use of different cell types. Indeed, vimentin is differentially phosphorylated by PKA/PKC in monocytes and macrophages, respectively, which may be linked to vimentin filament disassembly, resulting in cell surface localization and secretion of vimentin in macrophages [5,22,29]. The study of Mahesh *et al.* has further reported that ROS (reactive oxygen species) production is connected to VIM expression, *i.e.*, VIM expression is upregulated by ROS [22]. The authors proposed a model that involves an active mechanism of *M. tuberculosis* to limit ROS production. This in turn reduces VIM expression, which then supports intracellular persistence of *M. tuberculosis* in macrophages. One possible factor involved in *M. tuberculosis*-mediated ROS reduction and, subsequently, VIM downregulation could be ESAT-6, a substrate of the bacterial type VII secretion system. Another study has reported upregulation of vimentin in response to ROS in endothelial progenitor cells [30]. The apparent cross-talk between ROS production and vimentin expression needs further investigations. A recent study has shown the influence of another protein in the ROS-vimentin interconnection [31]; vimentin can bind to surface located dectin-1, a signaling pattern recognition receptor of the C-type lectin domain family. Vimentin can serve as an endogenous ligand for dectin-1 which results in activation of ROS production. This suggests that extracellular vimentin can induce oxidative stress.

The species *Mycobacterium avium* is an opportunistic pathogen and is linked to respiratory illness in immunocompromised patients, such as cystic fibrosis. The pathogen forms microaggregates on respiratory epithelial cells in the lung. In particular, binding of *M. avium* appears to involve vimentin on respiratory cells. Specifically, the bacterial protein microaggregate-binding protein 1 (MBP-1) can bind to host vimentin [23]. MBP-1 induces vimentin polymerization at the site of the bacterium-host cell interface. Taken together, the data suggests that vimentin mediates *M. avium* binding to and invasion of the host respiratory epithelium.

Another less well understood vimentin interaction has been reported for the skin bacterium *Propionibacterium acnes.* While comparing the bacterial impact on skin and prostate epithelial cells, we have found that *P. acnes* can efficiently invade prostate cells that express vimentin [24]. Vimentin depletion results in strongly reduced bacterial invasion and intracellular bacterial persistence. Interestingly, the immune response in *P. acnes*-challenged prostate cells is governed by NF-κB; depletion of vimentin drastically reduces the NF-κB-mediated immune response to *P. acnes* [24]. It needs to be explored if this finding is a consequence of reduced invasion of *P. acnes* into the host cell or of reduced interference of intracellular vimentin with immune signaling. These two roles of vimentin, *i.e.*, pathogen binding to the cell surface and mediation of intracellular innate immune signaling, might be combined. Interestingly, the presence of vimentin in acne-affected sebaceous follicles has been shown, which might be a response to injury and wound healing [25]. Vimentin expression might stimulate *P. acnes* binding and invasion into the injured skin epithelium and might go along with prolonged *P. acnes*-triggered inflammation as seen in inflammatory acne.

## 4. Conclusions

Here, we have summarized the current knowledge of vimentin interactions with microorganisms, focusing on bacterial pathogens. Also, several viruses can interact with vimentin in similar ways as bacterial species and similar cellular consequences have been reported. For example, vimentin can act as a virus receptor for the severe acute respiratory syndrome coronavirus (SARS-CoV) and for Enterovirus 71 [32,33].

The existing data has so far highlighted microorganism-vimentin interactions that partly depend on the localization of vimentin (Figure 1):

1. Surface-located vimentin has been identified as an attachment receptor for pathogen entry. The interaction between the microorganism and vimentin may be an initial weak, supportive attachment, to facilitate the interaction with other cell surface receptors, resulting in enhanced bacterial/viral endocytosis. Taken together, surface-located vimentin facilitates pathogen invasion. In addition, extracellular vimentin derived from activated macrophages has been detected in response to pro-inflammatory signaling [5]. Its function is less well understood, but it might be involved in pathogen trapping and killing. Most of these studies have been carried out in professional phagocytes, such as macrophages. Thus, pathogen binding and uptake is part of the defense system of these phagocytes to eliminate bacterial pathogens. Intracellular pathogens might have subverted this defense strategy to their own favor, resulting in intracellular persistence and protection from adaptive immunity. Taken together, surface-located vimentin as well as extracellular vimentin might be components of the host defense system to clear microbial infections.

2. Cytosolic vimentin is a component of the cytoskeleton. The intracellular pathogen, usually confined in a pathogen-containing vacuole, interferes with cytosolic vimentin in order to sustain an intercellular niche. Changes of this intracellular niche can go along with pathogen-mediated vimentin modifications or proteolysis to modulate vimentin filamentation and localization.

3. In addition, cytosolic vimentin has another role as a mediator of the innate immune response. In this regard, vimentin has been shown to be an endogenous ligand of at least three pattern recognition receptors (PRRs), *i.e.*, NOD2, Dectin-1, and NLPR3 [11,28,31]. In addition, other PRRs might interact with vimentin as well, such as RIG-I-like receptors that can sense viral infections [34]. Upon sensing of pathogens (via pathogen-associated molecular patterns), PRR signaling results in NF-κB or inflammasome activation, respectively, both defense responses in order to restrict microbial growth and dissemination. As such, vimentin as well as other components of the cytoskeleton are determinants of the host cell defense mechanism to eliminate intracellular pathogens [35]. To counteract PRR-mediated signaling, bacterial pathogens have evolved factors that interfere with the vimentin-inflammation axis. Such bacterial factors can exert post-translational modifications of vimentin by phosphorylation/dephosphorylation or ADP-ribosylation (and possibly other modifications) in order to modulate or intercept PRR signaling. This also implies that the role of vimentin not only depends on its localization but also on the filament size and on the presence/absence of certain post-translational marks. The success of the intracellular pathogen seems to depend, at least partly, on its ability to manipulate the vimentin network in order to suppress innate immunity and to sustain intracellular persistency.

Several open questions remain to be addressed before we can understand the exact role of vimentin in pathogen recognition and binding, PRR-mediated immune signaling, and the autophagy process in particular, as vimentin has also been shown to have a role in regulating autophagy [36]. Results from these studies form the basis for possible therapeutic interventions that target vimentin-mediated pathogen binding and/or the vimentin-inflammation axis.

## Figures and Tables

**Table 1:** Key findings of selected bacterial interactions with vimentin.

Bacterial Species	Host Cell	Interaction and Outcome	Virulence Factor	Reference
**Gram negative**				
*Escherichia coli*	endothelial cells	bacterial binding and invasion; vimentin interaction with NOD2; activation of NF-kB and ERK1/2	IbeA	[6,7,8,9,10,11]
*Salmonella enterica*	epithelial cells macrophages	vimentin recruitment to membrane ruffles; bacterial invasion; fixation of vacuole in juxtanuclear area	SptP	[12,13]
*Anaplasma phagocytophilum*	neutrophils endothelial cells	modulation of vimentin network; activation of ERK1/2 signaling	AptA	[14]
*Chlamydia trachomatis*	epithelial cells	vimentin remodeling and cleavage; de/stabilization of bacterial vacuole	CPAF	[15,16,17]
**Gram positive**				
*Streptococcus pyogenes*	epithelial cells macrophages muscle cells	bacterial binding; ADP ribosylation of vimentin leading to inhibition of vimentin filament formation; altered immune signaling; inflammasome (?)	SpyA	[18,19,20]
*Mycobacterium tuberculosis*	monocytes macrophages	modulation of vimentin expression; altered ROS production; bacterial persistence	ESAT-6?	[21,22]
*Mycobacterium avium*	epithelial cells	bacterial binding; vimentin polymerization	MBP-1	[23]
*Propionibacterium acnes*	epithelial cells	bacterial binding and invasion; modulation of immune signaling	?	[24,25]
